# New Developments and Opportunities of Microbiota in Treating Breast Cancers

**DOI:** 10.3389/fmicb.2022.818793

**Published:** 2022-05-12

**Authors:** Zihui Meng, Zixuan Ye, Pengrong Zhu, Jianguo Zhu, Shuguang Fang, Tianzhu Qiu, Yanan Li, Lijuan Meng

**Affiliations:** ^1^School of Food Science and Pharmaceutical Engineering, Nanjing Normal University, Nanjing, China; ^2^Wecare Probiotics Co., Ltd., Suzhou, China; ^3^Department of Oncology, First Affiliated Hospital of Nanjing Medical University, Nanjing, China; ^4^Department of Geriatric Oncology, First Affiliated Hospital of Nanjing Medical University, Nanjing, China

**Keywords:** breast cancer, tumor environment, microbiota, targeted therapy, immune

## Abstract

Despite the prevalence of breast cancer (BC), over half of BC cases are unrelated to known risk factors, which highlights the importance of uncovering more cancer-related factors. Currently, the microbiota has been proven to be a potent modulator of the tumor environment in BC, which regulates the immune balance in tumor-related networks. Through a large amount of data accumulation, the microbiota has shown many possibilities to reveal more insights into the development or control of BC. To expand the potential benefits of patients with BC, this study discusses the distribution profile and the effect mechanism of BC-related microbiota on tumors and further discusses its impact on different tumor therapies. Finally, we summarize the possibility of targeting microbiological therapies to improve BC treatment or in combination with other therapies.

## Introduction

In 2020, there were an estimated 2.3 million patients (11.7% of new cancer cases) diagnosed with breast cancer (BC). The Global Cancer Statistics 2020 report also indicated that BC has been the leading cause of cancer-associated death in women (Sung et al., 2021). According to the expression of the hormone receptors [estrogen (ER+), progesterone (PR+), and human epidermal growth factor receptor 2 (HER2 +)], BC is subclassified into five types: luminal A, luminal B, normal-like, HER2-enriched, “basal-like,” or triple-negative subtypes (TNBC) (Vuong et al., 2014). Clinically, chemotherapy, endocrinotherapy, and targeted therapy are the primary therapies against BC in addition to surgery and radiotherapy. Unfortunately, in addition to resistance and recurrence, these common therapies are always accompanied by severe side effects, which reduce the compliance of patients with BC. Due to the lack of endocrinotherapy or targeted therapy options, anti-PD-1 immunotherapies have received great attention in the TNBC. However, only a small number of patients can benefit from anti-PD-1 immunotherapy due to the heterogeneity of the tumor immune microenvironment. Therefore, more strategies are still required to improve the therapeutical effect of BC, especially novel therapeutic agents.

The microbiome is an important determinant of human health and disease, including cancer. The tumor microenvironment (TME) is a relatively independent and complex system composed of tumor cells, stromal cells, and various immune cells. Recent evidence indicates that distinct communities of microbiota inhabit tumor tissues in the body, including those previously considered “sterile,” such as breast, lung, or prostate tumors. Surprisingly, Nejman et al. (2020) found that BC had richer microbiota in the TME than other tumor types. Recent studies have indicated that microbiota, as a newly discovered modulator of TME, influences tumor progression, drug resistance, and response to therapy (Greathouse et al., 2020). With the progress of research, some specific bacteria and their metabolites were linked with estrogenic and pro-inflammatory signals, which are associated with an elevated risk of BC. However, the markers and profiles of microbiota exhibited great differences among patients with BC with different tumor stages, age, and menopausal statuses. Untangling this complex interplay of the microflora with BC progression and therapy could open new avenues for BC detection and management. To expand the potential benefits for patients, it is necessary to clarify the role of microbes in BC diseases.

This review discusses the microbiota profiles of patients with BC and emphasizes the effects of BC-related microbiota and their metabolites on tumor progression. We then summarize the distinct effects of microbiota on the BC therapies. Based on these studies, we further discuss the possibility of targeted microbiological therapies to improve BC treatment (Chadha et al., 2020).

## The Overall Microbiota Profiles of Patients With Breast Cancer

In recent years, there has been a great interest in characterizing microbiota associated with BC. The human microbiota colonizes different habitats of our body, including the mouth, eyes, nose, skin, urethra, gut, vagina, and others (Fernandez et al., 2018). Most studies to date have focused on breast microbiota (breast mammary gland microbiota and breast milk microbiota) and gut microbiota in patients with BC.

### The Microbiota Profiles of the Body Fluids of Patients With Breast Cancer

There are still few studies evaluating urine and oral rinse samples of patients with BC. For example, Wang et al. (2017) collected urine, oral rinse, and surgically resected breast tissues from 57 patients with BC and 21 healthy controls to evaluate the differences in microbiome profile. The results showed no significant differences in oral rinse samples. The abundance of Gram-positive organisms was increased in the urinary samples of BC, but researchers believe that the differences are driven by menopausal status and BMI rather than cancer status (Wang et al., 2017). This view is consistent with results obtained using urinary samples (Fuhrman et al., 2014; Goedert et al., 2015). Changes in the diversity of microbiota in body fluids should be validated in larger cohort studies.

### The Microbiota Profiles of Breast Cancer Tissues

The microbiota profile of BC tissues is closely associated with tumor development. Xuan et al. (2014) first identified the presence of microbes in breast tissues, which contain a diverse and unique community of bacteria and is different from bacteria found in other parts of the body. One potential source of breast tissue microbiota might be skin or the mouth since skin or oral bacteria travel through nipple-areolar orifices to enter breast tissue (Fernandez et al., 2018). Hieken et al. (2016) reported that dendritic cells may collect bacteria from mucosal tissues and then facilitate bacterial transport from the gut lumen to the breast, especially during pregnancy and lactation. In addition, intestinal bacteria might also damage the dense tissue in the intestinal wall and translocate to the blood (Gopalakrishnan et al., 2018a). Therefore, it stands to reason that bacteria may occasionally reach the breast tissue through the systemic circulation. As summarized in Table 1, multiple bacterial genera exhibited significant differences in relative abundance when stratified by breast tissue type (tumor, tumor-adjacent normal, high-risk, healthy control), cancer stage, grade, and histological subtype. In general, the microbiota in BC tissues is distinct not only from those of healthy tissues but also from those of tumor-adjacent normal tissues (Nackerdien, 2008; Yuan et al., 2011). The dominant bacterial phyla in BC mainly included Proteobacteria, Firmicutes, and Actinobacteria. Many reports also found that the families Pseudomonadaceae, Ruminococcaceae, Sphingomonadaceae, Alcaligenaceae, and Clostridia were decreased in tumor-adjacent normal tissues compared with BC tissues (Yatsunenko et al., 2012). The diversity of these bacteria was richer in tumor tissues than in adjacent normal breast tissues (Nejman et al., 2020). Similarly, Bacteroidetes, Comanaceae, Bacillus, and Staphylococcus were relatively more abundant in tumor-adjacent normal tissues than in healthy breast tissues (Nejman et al., 2020), suggesting that the microbiota gradually changed from a healthy state to a cancerous state. To clarify this difference, Yatsunenko et al. (2012) isolated Enterobacteriaceae that were enriched in tumor-adjacent normal tissues. When *Escherichia coli* was cultured with HeLa cells, it induced DNA-double-stranded breaks *in vitro*. Similarly, DNA damage was also induced in human intestinal organoids by pks-positive *E. coli*, which produces the genotoxic compound colibactin (Pleguezuelos-Manzano et al., 2020). This provided a detailed explanation for the possible carcinogen-initiated effect of microbiota in tumor-adjacent normal tissues (Yatsunenko et al., 2012; Noguti and Lee, 2019).

**TABLE 1:** Summary of studies analyzing the relationship of gut and breast microbiota and breast cancer.

References	Microbiome type	Sample type and size	Main results
Urbaniak et al. (2014)	Breast tissue	45 BC samples, 13 benign tumor samples and 23 normal breast samples	The most abundant taxa in all tissues in the Canadian samples were *Bacillus* (11.4%), *Acinetobacter* (10.0%), unclassified *Enterobacteriaceae* (8.3%), *Pseudomonas* (6.5%), *Staphylococcus* (6.5%), *Propionibacterium* (5.8%), unclassified *Comamonadaceae* (5.7%), unclassified *Gammaproteobacteria* (5.0%) and *Prevotella* (5.0%). In the Irish samples in all tissues the most abundant taxa were unclassified *Enterobacteriaceae* (30.8%), *Staphylococcus* (12.7%), *Listeria welshimeri* (12.1%), *Propionibacterium* (10.1%) and *Pseudomonas* (5.3%).
Urbaniak et al. (2016)	Breast tissue	60 BC samples, 11 benign tumor samples and 10 normal breast samples	Women with BC had higher relative abundances of *Bacillus*, *Staphylococcus*, *Enterobacteriaceae* (unclassified), *Comamondaceae* (unclassified), and *Bacteroidetes* (unclassified). Healthy patients had higher relative abundances of *Prevotella*, *Lactococcus*, *Streptococcus*, *Corynebacterium*, and *Micrococcus*.
Hieken et al. (2016)	Breast tissue	15 pairs of BC and 13 pairs of benign diseases	Increased relative abundance in the following low-abundant genera in the breast tissue of women with invasive breast cancer, including *Fusobacterium, Atopobium, Hydrogenophaga, Gluconacetobacter*, and *Lactobacillus*.
Banerjee et al. (2015)	Breast tissue	100 TNBC samples along with 17 matched, and 20 non-matched controls	The highest prevalence detected in TNBC was *Arcanobacterium* (75%), followed by *Brevundimonas, Sphingobacteria, Providencia, Prevotella, Brucella, Eschherichia, Actinomyces, Mobiluncus, Propiniobacteria, Geobacillus, Rothia, Peptinophilus*, and *Capnocytophaga.*
Xuan et al. (2014)	Breast tissue	20 ER-positive breast cancer and paired normal tissue	Five richest phyla in patients with breast cancer are Proteobacteria, Firmicutes, Actinobacteria, Bacteroidetes, and Verrucomicrobia. *Methylobacterium radiotolerans* is relatively enriched in tumor tissue, while the bacterium *Sphingomonas yanoikuyae* is relatively enriched in paired normal tissue.
Chan et al. (2016)	Nipple skin and nipple aspirate fluid	25 females with a history of BC 23 healthy females	The nipple skin microbiome from HC and BC was not significantly distinguishable by their community composition, their diversity, or their individual OTUs, indicating that the nipple skin microbiome is independent of breast cancer history. The genus *Alistipes* was only present in the NAF from BC, while an unclassified genus from the family *Sphingomonadaceaewas* was relatively more abundant in NAF from HC.
Wang et al. (2017)	Breast tissue	57 women with invasive breast cancer undergoing mastectomy and 21 healthy women	*Methylobacterium* decreased in patients with cancer.
Yazdi et al. (2016)	Breast tissue and lymph nodes	123 lymph nodes and adjacent breast tissue; 5 normal mastectomy samples	Methylobacterium Radio tolerance was increased in breast cancer and has a positive correlation with tumor stage.
Thompson et al. (2017)	Breast tissue	668 breast tumor tissues and 72 non-cancerous adjacent tissues	*Proteobacteria, Actinobacteria*, and *Firmicutes* were increased in the tumor tissues and *Actinobacteria* abundance increased in non-cancerous adjacent tissues.
Banerjee et al. (2018)	Breast tissue	50 BRER BC tissues, 34 BRHR BC tissues, 24 BRTP BC tissues, 40 TNBCBC tissues and 20 breast control samples	BRER: *Arcanobacterium, Bifidobacterium, Cardiobacterium, Citrobacter, Escherichia;* BRHR: *Streptococcus;* BRTP: *Bordetella, Campylobacter, Chlamydia, Chlamydophila, Legionella, Pasteurella;* BRTN: *Aerococcus, Arcobacter, Geobacillus, Orientia, Rothia.* :
Costantini et al. (2018)	Breast tissue	38 specimens of both tumor and healthy adjacent tissues from 16 patients	Proteobacteria, Firmicutes, Actinobacteria, and Bacteroidetes were the most abundant phyla in breast tissue.
Meng et al. (2018)	Breast tissue	22 benign samples and 72 malignant BC tissues	Propionicimonas and families Micrococcaceae, Caulobacteraceae, Rhodobacteraceae, Nocardioidaceae, Methylobacteriaceae enriched in malignant tissue
Smith et al. (2019)	Breast tissue	83 breast tissue samples (64 BC tissue samples with 11 adjacent breast tissue samples, 8 healthy breast tissue samples)	Phylum Proteobacteria was most abundant in normal, normal adjacent to tumor, and BC tissue was with fewer *Firmicutes*, *Bacteroidetes*, and *Actinobacteria*. Breast tissues from NHB women had a higher abundance of genus *Ralstonia* compared to NHW tumors, which could explain a portion of the breast cancer racial disparities. Analysis of tumor subtype revealed enrichment of family *Streptococcaceae* in TNBC. A higher abundance of genus *Bosea* increased with stage.
Tzeng et al. (2021)	Breast tissue	221 patients with breast cancer, 18 individuals predisposed to breast cancer, and 69 controls	Invasive ductal carcinoma (IDC): Tepidiphilus, Alkanindiges, and Stenotrophomonas; Invasive lobular carcinoma (ILC): *Peptostreptococcus, Micromonospora, Faecalibacterium*, and *Stenotrophomonas*; HER2 + tumors: *Cloacibacterium, PRD01a011B, Alloprevotella, Stakelama, Filibacter, Blastomonas, Anaerostipes* Triple-negative breast cancer (TNBC): *Azomonas*
Goedert et al. (2015)	Fecal samples	48 post-menopausal females with BC and 48 control patients	BC patients had *Clostridiaceae*, *Faecalibacterium*, *Ruminococcaceae*, and lower levels of *Dorea* and *Lachnospiraceae.*
Luu et al. (2017)	Fecal samples	31 patients with early-stage breast cancer	In overweight and obese patients, the number of total *Firmicutes, F. prausnit-zii, Blautia sp.*, and *E. lenta* bacteria was significantly lower than that found in the normal BMI group. The total number of *Bacteroidetes, Clostridium coccoides cluster, C. leptum cluster, F. prausnitzii*, and *Blautia sp.* were significantly higher in clinical stage groups II/III than in clinical stages 0/I, with higher percentages of *C. leptum* cluster, *F. prausnitzii*, and *Blautia* sp. in the clinical stage group II/III.
Goedert et al. (2015)	Fecal samples	48 post-menopausal females with BC and 48 control patients	Cases were more likely than controls to carry IgA-coated *Betaproteobacteria Parasutterella*, particularly IgA-coated Betaproteobacteria *Parasutterella excrementihominis.* Cases were less likely than controls to carry eight taxa including IgA-coated Firmicutes *Clostridiales, Ruminococcaceae, Oscillibacter*, IgA-non-coated Bacteroidetes *Alistipesindistinctus*, and six IgA-non-coated Firmicutes *Clostridiales* taxa including IgA-negative *Ruminococcus*.
Fruge et al. (2020)	Fecal samples	32 female BC patients	Women with breast cancer with higher body fat had lower *Akkermansiamuciniphila* numbers.
Zhu et al. (2018)	Fecal samples	18 premenopausal breast cancer patients, 25 premenopausal healthy controls, 44 postmenopausal breast cancer patients, and 46 postmenopausal healthy controls.	Relative species abundance in the gut microbiota did not differ significantly between premenopausal breast cancer patients and premenopausal controls. 38 species were enriched in postmenopausal patients, including *Escherichia coli*, *Klebsiella*, *Prevotellaamnii*, *Enterococcus gallinarum*, *Actinomyces* sp. *HPA0247*, *Shewanella putrefaciens*, and *Erwiniaamylovora*. 7 species were less abundant in postmenopausal patients, including *Eubacterium eligens* and *Lactobacillus vaginalis*.
Horigome et al. (2019)	Fecal samples	124 BC patients	An increased relative abundance of *Actinobacteria* was significantly associated with increased levels of DHA. The relative abundance of *Bacteroidetes* was negatively correlated with the levels of EPA, and that of *Actinobacteria* was positively correlated with the levels of DHA in participants without a history of chemotherapy. *Bifidobacterium* only among participants without a history of chemotherapy
Ma et al. (2020)	Fecal samples	25 BC patients and 25 patients with benign breast disease	The relative abundance of *Firmicutes* and *Bacteroidetes* were decreased, while the relative abundance of *verrucomicrobla*, *proteobacteria*, and *actinobacteria* was increased in the breast cancer group.

*BC, breast cancer; TNBC, triple-negative breast cancer; ER, estrogen receptor; NAF, nipple aspirate fluid; PR, progesterone receptor; BRER, estrogen receptor or progesterone positive; BRHR, human epidermal growth factor receptor 2 (HER2) positive; BRTP, estrogen, progesterone and HER2 receptor positive.*

In addition to the difference in microbes between tumor tissues, normal tissues, and adjacent tissues, different BC subtypes or tumors with different statuses were also found to exhibit unique microbiota profiles (Yatsunenko et al., 2012; Eisenhofer et al., 2019). As shown in Table 1, luminal A, luminal B, HER2 + BC, ER + positive BC, metastatic BC, and TNBC harbored differentially abundant microbiota. For instance, Tzeng and coworkers found that HER2 BC harbored a significantly higher abundance of *Cloacibacterium, Alloprevotella, PRD01a011B, Blastomonas, Stakelama, Filibacter*, and *Anaerostipes*, compared to HER2-negative tumors. Furthermore, the reduced abundance of *Oblitimonas* was associated with tumor metastatic potential (Ingman, 2019; Tzeng et al., 2021). However, it still remains difficult to analyze and compare the BC microbiota data from individual studies due to several objective reasons. In the future, a standard protocol for the analysis of breast-related microbiota should be developed. Based on consistent results, the associations between microbiota and clinical tumor stages could be accurately established, enabling us to assess whether this microbiota can be used as the markers of specific tumor types or status.

### The Intestinal Microbiota Profiles of Patients With Breast Cancer

Since the gastrointestinal tract is the main habitat of microorganisms in the human body, most studies focused on examining the characteristics of gut microbiota in patients with BC. In a case-control study, an increased risk of BC was first linked with microbiota dysbiosis (Velicer et al., 2004). Commensal dysbiosis was confirmed to be associated with tissue inflammation, myeloid recruitment, fibrosis, and dissemination of tumor cells (Buchta Rosean et al., 2019), which further identified the importance of gut microbiota balance for patients with BC (Table 1). First, the diversity of fecal microbiota was changed in patients with BC, including the higher levels of *Clostridiaceae, Faecalibacterium*, and *Ruminococcaceae* (Goedert et al., 2015). Another study compared the differences in gut microbiota between 11 patients with BC and 7 healthy controls and identified richer *Clostridia, Enterobacterium, Lactobacilli, Bacteroides*, and *E. coli* in the patients (Minelli et al., 1990). Moreover, the intestinal microbiota of patients with BC were remarkably different in different tumor stages. *Blautia* sp. was found to be associated with the most severe clinical stage and histological grade by influencing the metabolism of estrogen (Luu et al., 2017). In addition, the screening of microbial markers for the diagnosis of BC should also consider the pre/postmenopausal status of patients (Hou et al., 2021). Hou et al. (2021) found that *Bacteroides fragilis* was specifically present in young women of premenopausal statuses and *Klebsiella pneumoniae* in older women of postmenopausal status.

Among the specific gut microbiota, some species can promote the progression of BC, while others can suppress tumor growth or sensitize the cancer cells to antitumor therapy (Plottel and Blaser, 2011; Mani, 2017). The exact roles that gut microbiota play in BC progression and response to immunotherapy will be discussed in a later section.

## The Role of Distinct Microbiota in Breast Cancer Tumorigenesis and Metastasis

**FIGURE 1:**
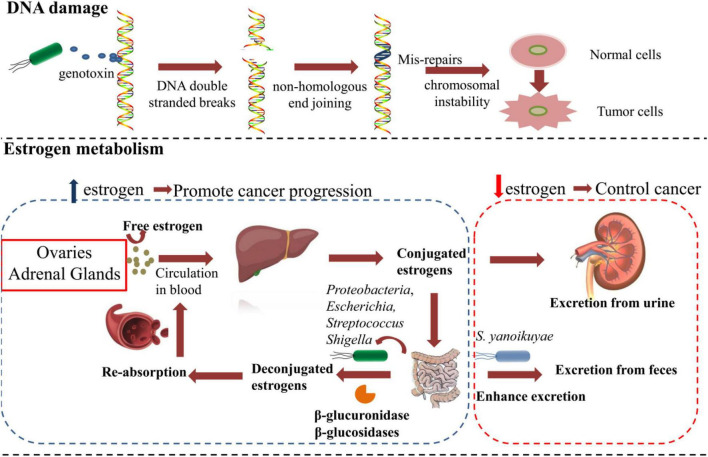
The microbiota affects breast cancer tumorigenesis and metastasis by DNA damage and regulating the estrogen metabolism.

**FIGURE 2:**
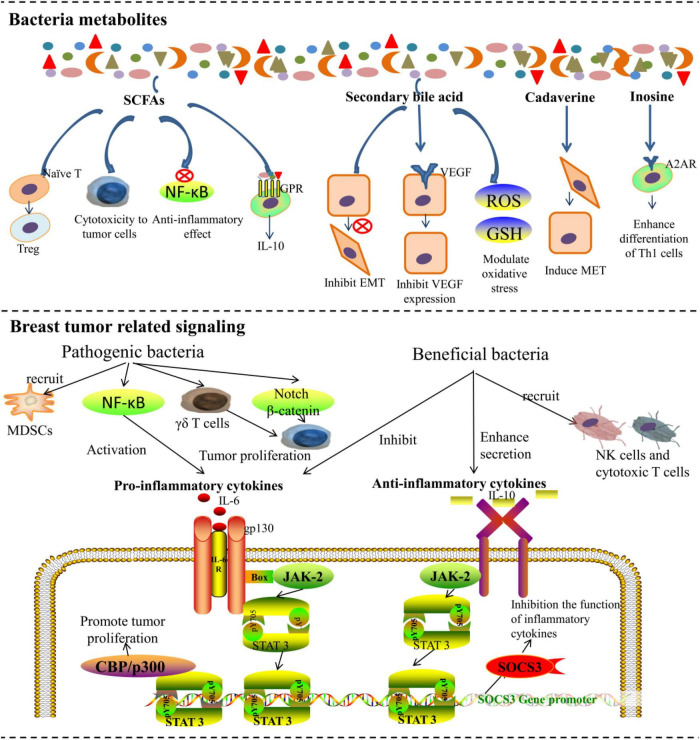
The microbiota affects breast cancer tumorigenesis and metastasis by metabolites and regulating tumor-related immune signaling networks.

Microbiota plays a complex role in the occurrence, development, and control of BC through multiple mechanisms. According to recent research and classification, distinct microbiota affects BC tumorigenesis and metastasis mainly in the following ways. First, the DNA damage or gene mutations caused by bacteria may promote the development of BC (Figure 1). Second, microbiota can influence the estrogen metabolism of patients with BC. Third, microbiota can produce metabolites that indirectly affect cancer development (Figure 2). Moreover, bacteria can directly affect immune signaling pathways involved in tumor-related networks (Laborda-Illanes et al., 2020; Alpuim Costa et al., 2021).

### DNA Damage Induced by Pathogenic Bacteria

DNA double-strand breaks (DSBs) are the most detrimental type of DNA damage, which is commonly caused by reactive oxygen species (ROS), genotoxic compounds, and radiation (Lees-Miller and Meek, 2003). The repair of DSBs through non-homologous end-joining is extremely error-prone and often leads to loss of bases at the site of damage (Lees-Miller and Meek, 2003). After the accumulation of faulty nucleotides in cells, the risk of genomic instability increases and eventually results in tumorigenesis. Studies have found that DSBs induced by certain strains of *E. coli* and *Helicobacter pylori* can lead to chromosomal instability after prolonged exposure (Cuevas-Ramos et al., 2010; Toller et al., 2011). *E. coli* strains of the B2 phylotype have the *pks* island, which encodes the production of genotoxic colibactin (Khanna and Jackson, 2001), which contributes to DSBs and the development of cancer. Urbaniak et al. (2016) found that certain strains of *E. coli* isolated from BC displayed the ability to cause DSBs. However, they proposed that the bacteria-induced DSBs may not be effective to promote cancer progression unless they occur in a genetically susceptible host (Urbaniak et al., 2016). How harmful bacteria promote BC tumorigenesis and progression through DSB-related mechanism requires further research.

### Effects of Bacteria on Estrogen Metabolism

A number of studies revealed a relationship between the microbial metabolome with BC tumorigenesis and metastasis (Ingman, 2019; Miko et al., 2019). Endogenous estrogen is the most important risk factor for the development of BC. Microbiota contributes to the reabsorption of free hormones and the metabolism of estrogen, which contributes to BC progression (Zacksenhaus et al., 2017; Gandhi and Das, 2019; Miko et al., 2019). Plottel and Blaser (2011) proposed a concept named estrobolome, which denotes the aggregate of enteric bacterial genes whose products are capable of metabolizing estrogens. Estrogens are synthesized mainly in ovaries before menopause and in adipose tissues in postmenopausal women (Parida and Sharma, 2019). Estrogens are then irreversibly hydroxylated in the liver and conjugated *via* glucuronidation and sulfonation. Most estrogens are excreted through urine or feces. However, a small but significant portion of conjugated estrogens can be deconjugated by gut bacteria that process β-glucuronidase activity and reabsorbed into the bloodstream (Kwa et al., 2016). Generally, the estrobolome could modulate estrogen metabolism *via* the enterohepatic circulation by virtue of bacterial β-glucuronidases and β-glucosidases, further affecting circulating and excretory estrogen levels (Kwa et al., 2016). Members of the phylum Proteobacteria, especially the genera *Escherichia* and *Shigella*, were reported to have β-glucuronidases activity and lead to estrogen upregulation (Alpuim Costa et al., 2021). Similarly, *Streptococcus pyogenes* was also reported to increase estrogen levels through β-glucuronidases activity in BC (Thompson et al., 2017). *Magnesium glucuron* in the gastrointestinal tract was found to promote reabsorption of estrogen, and the increased estrogen levels were closely related to the progression of BC (Jagannathan and Sharma, 2017). The abundance of *Bacillus* species was also elevated in patients with BC (Urbaniak et al., 2016). One report indicated that a *Bacillus cereus* strain, isolated from gingival plaque, metabolized the hormone progesterone into 5-alpha-pregnane-3,20-dione (5αP), which is known to enhance tumor progression by stimulating cell proliferation (Ojanotko-Harri et al., 1990; Wiebe et al., 2000; Wiebe, 2006). However, the concept of the estrobolome is mainly verified in postmenopausal patients with BC, and the evidence of the association with systemic estrogen in premenopausal women is still limited because of the variation of hormone levels during the menstrual cycle. Hou et al. (2021) found that the microbiota of premenopausal patients with BC was involved in the degradation of steroid-related aromatics and androstenedione, which is converted to estrogen. This upregulation of estrogen was similar with that in postmenopausal patients with BC.

Some beneficial bacteria regulate the metabolism of estrogens and reduce the risk of BC. For instance, *Sphingomonas yanoikuyae*, which was found to be relatively enriched in healthy controls, is a potentially protective factor because it enhanced the local metabolism and excretion of estrogens (Xuan et al., 2014). In addition, specific types of intestinal bacteria, such as *Coriobacteriaceae, Slackia*, *Adlercreutzia*, and *Ruminococcus*, are capable of metabolizing phytoestrogens (isoflavones and lignans), and converting them into active metabolites might protect against BC (Heinonen et al., 2001; Wang, 2002; Clavel et al., 2006; Cardona et al., 2013).

### The Effects of Bacterial Metabolites on Breast Cancer

The gut microbiota produced short-chain fatty acids (SCFAs), secondary bile acids, polyamines, and vitamins that affect cancer development. Previous studies showed that cadaverine, succinate, and p-cresol metabolites can also retard BC progression, indicating a non-negligible role of bacterial secondary metabolites (Ravnik et al., 2021).

At present, SCFAs are the most widely studied metabolites of microbiota. Compared with healthy premenopausal women, the abundance of SCFAs-producing bacteria and the key SCFA-producing enzymes were significantly reduced in premenopausal patients with BC. Although less is known about the exact mechanisms through which SCFAs exert their effects (He et al., 2021), it was found that SCFAs can directly affect apoptosis and invasion in BC cells (Salimi et al., 2017; Thirunavukkarasan et al., 2017). Propionic and acetic acids are produced by Bacteroidetes, while butyrate can be produced by *Roseburia inulinivorans* (Duncan et al., 2002; Scott et al., 2011) and *Firmicutes* (Mirzaei et al., 2021). *E. coli* KUB-36, which can produce 7 different SCFAs, was isolated from the intestines of a healthy human. *E. coli* KUB-36 demonstrated higher cytotoxicity in BC cells, which was attributed to its SCFA production (Nakkarach et al., 2021). Moreover, SCFAs such as butyrate have significant anti-inflammatory effects by inhibiting the activation of the nuclear factor-kB (NF-kB) pathway, which slows the tumor progression (Inan et al., 2000). Moreover, SCFAs can also interact with immune cells *via* G protein-coupled receptors (GPRs) to influence immunity, but their roles in BC need further investigation (Meijer et al., 2010). It is also interesting that the butyrate-producing *Faecalibacterium prausnitzii* was capable of reducing the content of inflammation-promoting cytokines, such as tumor-necrosis factor-alpha (TNF-a), interleukin (IL)-6, and IL-8 while inducing the increasement of regulatory T cells and stimulating macrophages to release anti-inflammatory cytokines such as IL-10 (Park et al., 2007; Vinolo et al., 2011).

Secondary bile acids are exclusively synthesized by microbiota (Inan et al., 2000) and are cytostatic to BC cells (Miko et al., 2018). Lithocholic acid, a secondary bile acid, was found to exert cytostatic effects and reduce the metastatic potential of BC cells by inhibiting the epithelial-mesenchymal transition (EMT) and vascular endothelial growth factor (VEGF) expression. It was also found to increase the antitumor immune response, oxidative phosphorylation, and the TCA cycle while modulating oxidative stress (Miko et al., 2018; Kovacs et al., 2019a). Lithocholic acid is synthesized by *B. fragilis, Bacteroides intestinalis, Clostridium scindens, Clostridium sordellii, Clostridium hylemonae*, and *E. coli* (Kovacs et al., 2020). Cadaverine, another metabolite of microbiota, was found to induce mesenchymal-to-epithelial (MET) transition through trace amino acid receptors and finally reduce BC invasion. Bacterial production is the major source of cadaverine. The main cadaverine-producing bacteria include *Aeromonas veronii, Clostridium perfringens, E. coli, Edwardsiella tarda, Hafnia alvei, Raoultella ornithinolytica, Staphylococcus*, and *Streptomyces species* (Kovacs et al., 2019b). Recently, Wang et al. (2022) found that Clostridiales and the related metabolite trimethylamine N-oxide (TMAO) were more abundant in tumors with an activated immune microenvironment, which could activate the endoplasmic reticulum stress kinase PERK and enhance CD8 + T cell-mediated antitumor immunity in TNBC *in vivo*. In other tumor models, it was also found that inosine produced by *Bifidobacterium pseudolongum* can promote Th1 cell differentiation and enhance the effect of immunotherapy mediated by T cell-specific adenosine 2A receptor (A2AR) signaling (Mager et al., 2020).

These studies suggest that bacterial metabolites can have antitumor effects after clarifying the connection between probiotic bacterial metabolites and BC. Furthermore, exogenous supplementation of specific immune-activating metabolites can be used as a potential therapeutic strategy to improve the efficacy of immunotherapy for advanced BC.

### Bacterial Immune Signaling Pathways Involved in Tumor-Related Networks

Buchta Rosean et al. (2019) first demonstrated that targeted disruption of the gut microbiota could promote cancer metastasis in a mouse model of hormone receptor-positive BC. They pretreated mice with a broad-spectrum cocktail of antibiotics to cause commensal dysbiosis and then observed that the abundance of circulating tumor cells was increased in antibiotic-treated mice, resulting in subsequent spread to lymph nodes and lungs (Buchta Rosean et al., 2019). Researchers analyzed the cytokines and chemokines in the serum and mammary glands prior to tumor initiation. The results showed that BC metastasis was promoted by early inflammation and enhanced myeloid infiltration as a result of commensal dysbiosis. The impact of commensal dysbiosis on BC metastasis sheds light on the relationship between gut microbiota and BC metastasis. Notably, gut and breast microbiota might also modulate the tumor microenvironment. Mucous membranes are natural barriers to bacteria. Once the barrier function is impaired, pathogenic bacteria will invade the host mucosa. Pathogenic bacteria release pathogen-associated molecular patterns (PAMPs), which are recognized by Toll-like receptors, and can activate the NF-κB pathway, leading to the release of pro-inflammatory cytokines to trigger inflammation (Laborda-Illanes et al., 2020). Furthermore, pathogenic bacteria can also block antitumor immunity by recruiting myeloid-derived suppressor cells into the tumor environment and upregulating galectin-1 expression in tumor-associated γδ T cells. Additionally, interactions between bacteria and immune cells can also trigger inflammation (Rutkowski et al., 2015). Marwaha et al. (2020) proposed a hypothesis that the inflammation caused by bacterial infiltration disturbs the stem cell hierarchy and finally accelerates the progression of BC.

Other signaling pathways involved in BC tumorigenesis and metastasis have also been evaluated. Specific pathogenic bacteria were found to play a crucial role in the progression of tumors, which may be applied in the early diagnosis of BC, as well as the identification of a biomarker for cancer prognosis and tumor metabolic processes (Ou-Yang et al., 2007). Recently, Parida et al. (2021) reported a carcinogenic strain of *B. fragilis* from the colon that promotes breast tumorigenesis and metastatic progression *via* the Notch and β-catenin signaling pathways. *B. fragilis* was identified in reported clinical data and was found in cancerous breast tissue. Then, researchers studied its function in BC tumorigenesis and metastasis using a mammary intraductal model (MIND). Entero-toxigenic *B. fragilis* (ETBF) was found to cause mammary epithelial hyperplasia by releasing *B. fragilis* toxin (BFT). BFT was found to mediate cell migration and invasion by activating the β-catenin and Notch pathways, which resulted in a highly migratory and invasive phenotype in BC cells (Parida et al., 2021). *Fusobacterium nucleatum*, a confirmed carcinogen in colon cancer, was recently found to translocate to breast tumors through the blood *via* recognition of Gal-GalNAc by Fap2 (Parhi et al., 2020). Parhi et al. (2020) found that *F. nucleatum* was not only enriched in primary mammary tumors but also in lung metastases in a mouse model. Mice incubated with *F. nucleatum* had a higher metastasis burden than controls, which indicated that metastasis was caused by *F. nucleatum*. In addition to the above bacteria, *Peptostreptococcus* may also contribute to tumor progression in patients with BC (Yaghoubi et al., 2020). Strains of *Peptostreptococcus* (e.g., *Peptostreptococcus asaccharolyticus* and *Peptostreptococcus prevotii, Streptococcus pneumonia*) were found to promote the synthesis of ROS and increase intracellular cholesterol levels by stimulating Toll-like receptors 2 and 4 (TLR-2 and TLR-4) (Yuan et al., 2008). The increase in ROS further leads to an oxidant/antioxidant imbalance and eventually promotes tumorigenesis. Moreover, the cumulative increase in cholesterol levels was also found to enhance tumor proliferation (Batetta et al., 1999). However, the carcinogenic mechanisms of other pathogens, such as *Staphylococcus, Parvimonas*, and *B. cereus*, have yet to be explored.

Notably, there are also beneficial bacteria that exert an antitumor role through various immunomodulatory pathways. For example, *Faecalibacterium* was found to suppress the proliferation and induce the apoptosis of BC cells by inhibiting the secretion of IL-6 and phosphorylation of JAK2/STAT3 (Yaghoubi et al., 2020). Battal et al. (2014) also found that *Faecalibacterium prausnitzii* increased the secretion of extracellular vesicles, upregulated the secretion of anti-inflammatory cytokines (TGF-β2, IL-10, and IL-1a), and downregulated the secretion of pro-inflammatory cytokines (TNF-α and IL-6) in lung cancer and colorectal cancer. The function of immune cells such as natural killer (NK) cells and T cells is closely associated with the incidence of BC (Strayer et al., 1984; Imai et al., 2000). It is assumed that the functionality of immune cells is impaired in the TME. Bacteria that restore the function of immune cells are therefore crucial to tumor suppression. The species that are less abundant in BC (e.g., *Lactococcus* and *Streptococcus*) were also shown to activate murine splenic NK cells to prevent tumor growth (Kosaka et al., 2012). However, studies focusing on this aspect are still relatively limited, and the effects of different strains, as well as their balance, on the immune system still need more research and support.

## Role of the Microbiota in the Therapy Response of Breast Cancer

### Chemotherapy

Intestinal microbiota was shown to influence the efficacy and side effects of chemotherapy. For instance, paclitaxel (PTX) is commonly used to treat patients with primary BC, but PTX monotherapy causes intestinal dysbiosis. A report indicated that supplementation with the fungus *Ganoderma lucidum* was capable of ameliorating the dysbiosis caused by PTX, which significantly promoted the compliance of patients (Su et al., 2018). Neoadjuvant chemotherapy comprising a combination of anthracycline, an alkylating agent, and taxanes was found to increase the tumor proportional abundance of *Pseudomonas*, which was proved to have potent immunomodulatory effects and directly impact BC cell proliferative signaling (Chiba et al., 2020). The microbiota may also affect the efficacy of chemotherapy by metabolizing xenobiotic chemotherapy drugs (Sampsell et al., 2020). For instance, a diet enriched with *E. coli* OP50 increased the efficiency of 5-fluoro-20-deoxyuridine in the nematode *Caenorhabditis elegans*, whereas a diet enriched with *Comamonas* increased the response of camptothecin (Garcia-Gonzalez et al., 2017). The cytotoxic effects observed in *C. elegans* are dependent on bacterial ribonucleotide metabolism (Garcia-Gonzalez et al., 2017). The gut microbiota may also affect the side effects of chemotherapy. Beta-glucuronidase (GUS) enzymes, encoded by both humans and microbes, such as *Clostridium perfringens*, *Streptococcus agalactiae*, and *B. fragilis*, play important roles in chemotherapy by metabolizing certain drugs (Pollet et al., 2017). For example, an inactive metabolite of irinotecancan can be reactivated by β-glucuronidase, leading to severe drug effects (Ding et al., 2018). Gut microbiota has also been suggested to be closely associated with 5-fluorouracil induced mucositis (Li et al., 2017). Moreover, 5-fluorouracil was found to alter both gut microbiota and inflammatory cytokine/chemokine profiles, which were accompanied by mucosal barrier disruption and activation of inflammatory signaling pathways. Fecal transplantation alleviated gastrointestinal mucositis induced by 5-fluorouracil (Li et al., 2017). Therefore, we have reason to speculate that we could improve the efficiency and reduce the side effects of chemotherapy by regulating the gut microbiota.

### Immunotherapy

The relationship between immunotherapy and microorganisms has been studied extensively in tumors other than BC, such as pancreatic cancer, colon cancer, prostate cancer, and melanoma, and has been summarized in many reviews (Sampsell et al., 2020). However, immunotherapy is not widely available for BC. In recent years, immunotherapy such as anti-PD-1 inhibitors has become a promising treatment for patients with TNBC. However, the clinical data showed that the patients still had only limited benefits from this type of immunotherapy (Schmid et al., 2018; Sternschuss et al., 2021). Atezolizumab plus nab-paclitaxel prolonged the survival time of patients with metastatic triple-negative BC in the PD-L1 + subgroup (Schmid et al., 2018). Multiple primary studies demonstrated that the efficacy of immunotherapy can be attributed to specific microbiota (Gopalakrishnan et al., 2018b; Routy et al., 2018). Routy et al. (2018) collected samples from patients with lung and kidney cancer treated with immunotherapeutic PD-1 inhibitors and found that patients who had low levels of *Akkermansia muciniphila* were less responsive, which indicated that bacteria supplementation may restore the response to immunotherapy. A recent study found that *B. pseudolongum* produces inosine, which modulates responses to anti-CD47 immunotherapy (Mager et al., 2020). After inosine administration, intratumoral IFN-γ + CD4 + and IFN-γ + CD8 + T cell infiltration increased, thus enhancing the efficacy of checkpoint blockade immunotherapy (Mager et al., 2020). Other studies also revealed that immunotherapy works in part by recruiting key immune cells to the tumor site (Sivan et al., 2015; Vivarelli et al., 2019). Currently, the most common bacteria associated with favorable responses of various cancers to immunotherapy are Clostridiales, *Faecalibacterium*, Ruminococcaceae, *B. fragilis, Akkermansia muciniphila, bifidobacteria, enterococci*, and *Collinsella*, in various types of cancer. Among them, Wang et al. found that the proportion of *Clostridium* subtypes in the breast microenvironment was significantly higher than that of other subtypes. Moreover, these microbiota produced trimethylamine oxide to induce GSDME-mediated pyroptosis of BC cells and recruit CD8 + T cells in the microenvironment. This activated immune environment significantly promotes the response to immunotherapy (Wang et al., 2022). Pretreatment optimization of the gut or tumor microbiota may be a viable strategy for immunotherapy sensitization. However, more research on the mechanisms of microbial influence on the response to immune checkpoint inhibitors is needed in patients with BC.

### Other Therapies

As described above, standard BC treatments also include surgery, radiotherapy, hormone therapy, and targeted therapy. Hormone receptor-positive BC is often treated with hormone therapies that aim at lowering the amount of estrogen in the body or inhibiting the action of estrogen on BC cells. The effects of microbiota on estrogen have been summarized in the “Effects of bacteria on estrogen metabolism” section. Radiotherapy is often used to decrease the BC tumor burden and prevent a recurrence. In both male and female mice, changes in the intestinal bacterial communities can influence the radiosensitivity of tumors, and the gavage of fecal microbiota can help protect against radiation-induced side effects (Cui et al., 2017). Fecal microbiota transplantation (FMT) increased the survival rate by correcting gastrointestinal tract function and intestinal epithelial integrity in an irradiated mouse model (Cui et al., 2017). However, studies addressing distinct effects of microbiota on radiotherapy in BC are rare.

## Microbiota-Based Strategies for Breast Cancer Treatment

There is accumulating evidence that bacteria in the intestinal tract or tumor area influence oncogenesis, tumor progression, or response to anticancer therapy in BC. Microbiota is increasingly recognized for their influence on antitumor immunity, as well as therapeutic responses to cancer treatment. These strategies also provide a new angle to improve the therapeutic outcomes in BC, especially triple-negative or advanced recurrent BC.

In addition, therapeutics for the modulation of microbiota mostly initiate their antitumor effect by activating innate and adaptive antitumor immune responses or reversing immune suppression in the TME, which could be applied as adjuvant therapy to achieve enhanced antitumor effects. However, the function and action sites of various bacteria might be different or even have opposite effects. Therefore, undifferentiated regulation of the entire microbiota may result in a lack of response or unacceptable toxic effects. Precise modulation of tumor-related microbiota is the key for achieving improved safety and efficacy. As discussed below, the regulation of the bacterial community mainly includes the selective killing of tumor-promoting bacteria or the precise delivery of antitumor bacteria, which all aim at improving the therapeutic index when used alone or in combination with other antitumor approaches. Currently, several measures can be used to modify the gut microbiota or tumor microbiota for enhanced anticancer therapy, such as replenishing antitumor bacteria using fecal transplant, probiotics, prebiotics, and postbiotics, as well as killing the cancer-promoting bacteria using antibiotics, phage therapy, or other therapy. Additionally, the precise delivery of beneficial bacteria into specific sites can also be considered a precision strategy.

### Precise Supplementation of Antitumor Probiotics

Many reports have outlined potential strategies to modulate microbiota with increased antitumor effect, focusing on fecal microbiota transplants, probiotics, diet, and prebiotics. Supplementation of live bacteria has been extensively investigated in cancer therapy. For BC, supplementation of antitumor bacteria also may play a key role in regulating the tumor immune environment and enhancing the therapeutic effect. Several trials of fecal transplants from complete responders have been studied in patients with ICB therapy, showing increasingly positive antitumor responses in melanoma, colon cancer, and pancreatic cancer. However, there are no clinical studies on fecal bacteria transplantation in patients with BC. It seems that we are at the first step of determining the effect of fecal microbiota in patients with BC, which requires more development before the next step of fecal transplant. Here, we focused on the application of probiotic supplements in BC therapy.

Compared with colorectal and liver cancer, fewer reports are available on the effects of probiotics on cancer suppression in BC, especially in the clinic. In a preclinical study, researchers found that daily consumption of lactic acid bacteria (LAB) may decrease the risk of BC (Tannock and Rotin, 1989). Lakritz et al. (2014) further demonstrated that *Lactobacillus reuteri* was sufficient to reduce mammary neoplasia through the protective mechanism of CD4 + CD25 + lymphocytes against BC. Furthermore, administrating fermented milk that contains *L. acidophilus*, *L. bulgaricus, Streptococcus lactis*, or *Bifidobacteria* could inhibit the growth of ER + BC in animal models (Ohta et al., 2000; Chang et al., 2002; Takagi et al., 2015). Several probiotics were reported to secrete proteins with anti-BC and apoptosis-inducing effects on MCF-7 cells (Pourbaferani et al., 2021), including lactic acid bacteria and bifidobacterium. They can also produce exopolysaccharides (EPS) to inhibit the proliferation of BC cells *via* various mechanisms, including inducing cell cycle arrest or apoptosis, as well as having antimutagenic, antioxidative, or anti-inflammatory effects (Wu et al., 2021). Moreover, riboflavin-producing lactic acid bacteria were reported to prevent the interruption of conventional chemotherapy by reducing undesirable side effects (Levit et al., 2021) for patients with BC. However, due to the low probability that probiotics can interact directly with BC cells, the evaluation of probiotics and their metabolites was mostly performed *in vitro*. Recently, probiotics were also tested *in vivo* for more accurate results. Méndez Utz et al. (2021) found that probiotic *Lactobacillus casei* CRL431 could reduce the side effects of capecitabine and enhance its antitumor/antimetastatic effects by improving the host’s immune response and decreasing the immunosuppressive cytokines (IL-6 and IL-10) in a mouse model of BC. This further suggests that probiotics can be used as adjuvant therapy to consolidate the antitumor effect of chemotherapy and radiotherapy.

Probiotics were found to influence host immunity to maintain long-term therapeutic effects. In addition to the direct ancillary anticancer effects, probiotics also play an important role in preventing tumor metastasis or recurrence, resulting in a more favorable prognosis for patients with BC. Several studies have reported the effect of probiotics in regulating the levels of cytokines, such as IL-12, IL-4, IFN, and TGF-β, to inhibit tumor growth in BC tumor-bearing mice (Yazdi et al., 2010; Maroof et al., 2012). As an effective antitumor factor, IL-12 was confirmed to enhance the response to cancer immunotherapy (Lasek et al., 2014). Hence, specific probiotics could act as a great immune adjuvant for combined cancer therapy. Raji Lahiji et al. (2021) performed a randomized clinical trial among 76 overweight or obese postmenopausal women with a history of hormone-receptor-positive BC. Compared with placebo, the probiotic supplementation contributed to a significant decline in adiponectin, TNF-α, and high-sensitivity C-reactive protein (hs-CRP), indicating a reduced risk of recurrence (Raji Lahiji et al., 2021). Another study revealed that a unique probiotic “kefir” reduced tumor growth and metastasis of BC by stimulating and modulating T helper cells. Currently, there are only nine clinical studies on the effects of probiotics in BC, mostly focusing on examining the effect of probiotics on the tumor microbiota and gut microbiota of patients with BC. Generally, clinical data that support the beneficial effects of probiotics in combating BC are still limited, with studies mainly in the preclinical stage and needing further long-term validation.

For the broader application of probiotics in precise cancer therapy, the antitumor mechanism and effect of probiotics in patients with BC still need to be studied. Clarifying the specific mechanism of the anticancer effects or reduction of side effects is the premise for the popularization and application of probiotics in BC therapy. In addition, other challenges of probiotic application *in vivo*, such as low activity in the gastrointestinal tract and low adhesion to the intestinal mucosa, further hinder their therapeutic effect. Additionally, providing precise delivery systems to guarantee the specific contact with the intestinal mucosa is also essential for accurate modulation, further enhancing the regulation of the TME of BC.

### Therapies Targeting Tumor Microbiota to Potentiate Antitumor Effects

Along with the increased discovery of the function of bacteria inside the TME of BC, the specific colonizing bacteria have attracted much attention as a target in cancer therapy. As summarized above, *B. fragilis* and *Clostridium nucleatum* contribute to BC metastasis and immune escape, so more efforts to kill this kind of tumor-related microbiota for satisfactory tumor therapy have highlighted the possibility of selectively editing intratumoral microbiota. This strategy provided a new combination regimen to modulate tumor immune microenvironment (TIME) and improve the therapeutic effect of standard treatment for BC.

The microbiota inside the BC tumor and intestinal system play different roles in tumor progression, either beneficial or detrimental. For example, *Fusobacterium* and *B. fragilis* have been shown to enter the tumor tissue of BC, promote tumor growth and metastasis, result in a suppressive TME, and induce resistance to antitumor therapy. However, other specific bacterial species found in breast tissues, such as *Lactococcus lactis*, have also been shown to increase the expression of anti-inflammatory response pathways or activate NK cells to control tumor growth. Moreover, the metabolites of *Lactococcus lactis*, especially SCFAs, could further enhance antitumor immune responses and inhibit cancer development. Therefore, it is challenging to determine whether broad-spectrum antibiotics could produce beneficial antitumor effects. Antibiotics are commonly used to reduce the risk of infection in postsurgical or immunocompromised patients with BC. However, according to the clinical data (Ahmed et al., 2018), the adjuvant broad-spectrum antibiotic therapy has been associated with a disruption of intestinal balance, resulting in poor outcomes for patients because of the severe dysregulation and broken homeostasis (Geller et al., 2017). Since large clinical studies indicated that the use of broad-spectrum antibiotics is closely related to the increased risk of BC, it is important to clarify the effects of antibiotics on the pathways being regulated by the host microbiota to achieve a better antitumor effect. To optimize the effect of this strategy, the idea of “personalized antibiotics” has been recently proposed to selectively kill tumor-associated harmful bacteria. For example, the Gammaproteobacteria in tumor tissue can degrade gemcitabine into an inactive form by secreting cytidine deaminase. Accordingly, adjuvant treatment with the antibiotic ciprofloxacin greatly increased the antitumor effect of gemcitabine (Geller et al., 2017). Regardless of their promising antitumor effects, these “customized antibiotics” also face many challenges. First, there are currently no antibiotics that target only one type of bacteria, and developing highly specific antibiotics is very difficult. Moreover, most antibiotics are affected by the development of bacterial resistance, preventing their long-term efficiency. Therefore, more precise approaches to target and modulate specific intestinal/intratumoral microbiota are essential.

In the future, many precise strategies should be investigated to remove the harmful bacteria in BC tumors, including specific antimicrobial peptides, antimicrobial materials, and bacteriophages. Phage-guided systems are considered to be a potential delivery vehicle for microbiota modulatory therapies. Phage display technology has been widely used in the field of tumor therapy and diagnoses, such as screening tumor-targeting peptides, targeted delivery of chemotherapeutic drugs, and the preparation of antitumor antibodies (Ferreira et al., 2019). Recent studies revealed that phage therapy could also remove the tumor-promoting bacteria *via* accurate species-specific mechanisms (Dong et al., 2020). Although targeting tumor-promoting bacteria or the communication between commensal microbiota and host cells was mostly restricted to gastrointestinal tumors due to their clear relationship with bacteria, with the discovery of the roles of bacteria and communication mechanisms in BC, microbiota-targeting therapies could be adapted for various types of BC in the future.

## Conclusion and Future Directions

The role of microbiota in BC is like a double-edged sword. Generally, compared with that in gastrointestinal and pancreatic cancers, the development of the relationship between host microbiota and BC is still in the early stage, which needs more clinical and preclinical investigation. Although the correlation between some bacteria and tumor development or therapeutic response has been well established, the mechanisms that bacteria use to communicate with cells or tissues should be further investigated as this would help in the design of more specific and efficient regimens. For instance, as *F. nucleatum* enters into tumors through recognition of Gal-GalNAc by Fap2, a novel method like Fap2 antibodies or Gal/GalNAc antagonists can be considered as an alternative to antibiotics. In addition, the composition and distribution of host microbiota may be considered a predictive biomarker for BC prognosis and subsequent treatment regimen. In contrast, with the discovery of bacteria–drug interactions, the bacterial composition can also be used as a reference for personalized treatment for patients with cancer, and even bacteria-modified regimens can be used as adjuncts to conventional treatment of cancer. To support these goals, the large-scale screening of distinct bacteria in clinical patients needs to be carried out. Furthermore, more validation work is needed on animal models, as well as clinical trials, to provide evidence on the status of bacteria in the treatment of BC.

## Author Contributions

ZM, ZY, YL, TQ, and LM initiated and conceived the review. ZM, ZY, and JZ participated in selecting and reviewing literature data. SF and PZ analyzed based on the literature and wrote the literature. TQ, YL, and LM supervised and revised the manuscript. All authors read and approved the final manuscript.

## Conflict of Interest

JZ and SF were employed by the Wecare Probiotics Co., Ltd. The remaining authors declare that the research was conducted in the absence of any commercial or financial relationships that could be construed as a potential conflict of interest.

## Publisher’s Note

All claims expressed in this article are solely those of the authors and do not necessarily represent those of their affiliated organizations, or those of the publisher, the editors and the reviewers. Any product that may be evaluated in this article, or claim that may be made by its manufacturer, is not guaranteed or endorsed by the publisher.
